# Selection plays the hand it was dealt: evidence that human adaptation commonly targets standing genetic variation

**DOI:** 10.1186/s13059-017-1280-5

**Published:** 2017-08-01

**Authors:** Rajiv C. McCoy, Joshua M. Akey

**Affiliations:** Department of Genome Sciences, University of Washington, Seattle, WA 98195 USA

## Abstract

Using a powerful machine learning approach, a recent study of human genomes has revealed widespread footprints of recent positive selection on standing genetic variation.

Adaptation by natural selection is responsible for the extraordinary diversity of life on Earth, as well as the striking matching of organisms to their environments. Yet the role of adaptation in recent human evolution remains controversial. Schrider and Kern [1] recently applied a novel machine learning approach to systematically evaluate the evidence for positive selection in humans. The authors argue that adaptation is pervasive, but commonly targets standing variation and leaves subtle genomic footprints that have not been detectable by previous methods. This work challenges long-standing assumptions about human evolution.

## Controversy surrounding the role of positive selection in shaping genetic variation

Understanding the frequency, mechanisms, and specific targets of past adaptation constitutes a central goal of human evolutionary biology. Facilitated by rapid advances in DNA sequencing, large-scale scans for positive selection have revealed several convincing candidates including mutations in *LCT*, which confers lactase persistence in European and African populations, and mutations in *EDAR*, which influences skin and hair phenotypes in East Asian populations. Such episodes of positive selection reduce variation in a wide genomic region linked to the selected site, thereby generating a signature termed a ‘selective sweep’.

**Fig 1:**
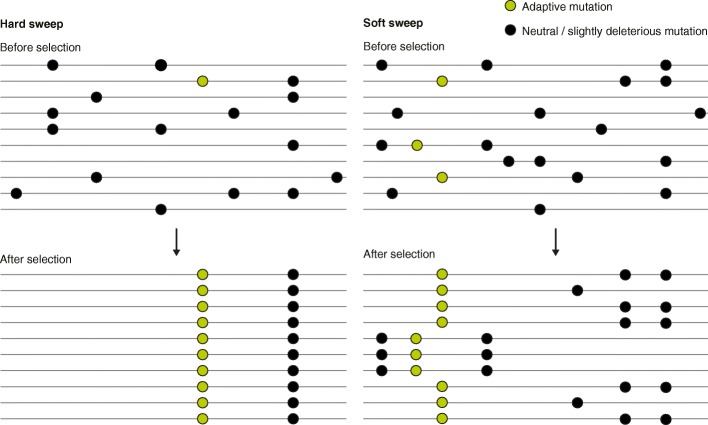
Schematic describing the impacts of hard and soft selective sweeps on patterns of linked genetic variation. Each row depicts an individual haploid genome. Adaptive mutations are colored in *green*, while neutral or slightly deleterious mutations are colored in *black*. Alleles that match the reference genome are not depicted. A hard selective sweep (*left panel*) involves a single adaptive mutation that arises de novo and subsequently increases in frequency, carrying with it neutral and slightly deleterious variants to which it is linked. By contrast, a soft selective sweep involves selection on either standing variation or recurrent de novo mutations, such that adaptive alleles are present on multiple distinct haplotypes. These adaptive mutations may or may not occur in the same genomic position. All haplotypes carrying an adaptive allele simultaneously sweep to higher frequency, leading to only modest reductions in levels of nearby genetic diversity

Yet aside from a few well-characterized examples, there remains considerable debate about the tempo and mode of selective sweeps in humans and about the proportion of genome-wide variation that is influenced by linked selection [2–5]. Scans for positive selection have traditionally targeted long, high-frequency, derived haplotypes in regions of reduced genetic variation. These signatures are expected under a classic model of a ‘hard selective sweep’. Such a scenario is expected when the supply of adaptive mutations is limited. Eventually, a single beneficial mutation may arise de novo, establish, and then increase rapidly to fixation. Neutral and slightly deleterious variants may simultaneously ‘hitchhike’ to high frequency through linkage to the adaptive allele. The result is a drastic reduction in variation both at the selected site and in the surrounding genomic region (Fig. 1)—a signature that can persist for thousands of generations and is only slowly eroded by recombination. An alternative mode of adaptation is that of a ‘soft selective sweep’, where multiple beneficial haplotypes simultaneously increase in frequency [6] (Fig. 1). The footprints of soft sweeps are expected to be more muted and to include modest skews in the frequency spectrum along with elevation of linkage disequilibrium. Soft sweeps may arise either by selection on standing variation or through the recurrence of adaptive mutations prior to sweep completion. Under close examination, several classic examples of selective sweeps appear to fall into the soft-sweep category. Selection on *LCT*, for example, targeted independent adaptive mutations in Africa versus Europe, such that the sweep appears hard at the local level but is soft at the global level.

## A novel approach for detecting positive selection

These theoretical and empirical findings about the complexity of selection signatures have prompted the development of methods that are sensitive to both hard and soft sweeps and capable of distinguishing between the two. Two such statistics, termed *H*_*12*_ and *H*_*2*_/*H*_*1*_, were recently developed for this purpose and revealed a striking predominance of soft selective sweeps in data from *Drosophila melanogaster* [7]. The relevance to human populations has, however, remained an open question.

To address this question, Schrider and Kern [1] used a sophisticated machine learning method that they previously developed for the robust detection of selective sweeps. Their approach, termed soft/hard inference through classification (S/HIC) [8], uses supervised machine learning to leverage multiple sweep signatures including reduced haplotype diversity, skews in the allele frequency spectrum, and increased linkage disequilibrium in regions flanking the selected site. Although any particular signal may be subtle or absent at any individual locus under selection (especially for soft sweeps), the combination of signals together provides sufficient information for S/HIC to confidently annotate each genomic window as hard, hard-linked, soft, soft-linked, or neutrally evolving. The method had already been shown to be relatively robust to assumptions about demographic history [8]. This is particularly important in human populations, which are thought to have experienced extreme bottlenecks, complex patterns of movement and replacement, and extensive gene flow, with substantial uncertainty in each of these parameters.

The authors applied their method to population genomic data from six populations that have relatively low levels of historical admixture: two West African, one East African, one European, one East Asian, and one American. Across all populations, they identified a total of 1927 distinct selective sweeps, including 519 (26.9%) loci identified in previous scans, as well as 1408 novel hits. Notably, most sweeps were either population-specific or shared among a subset of a few populations, potentially reflecting the importance of local adaptation. Because signals diminish over time, however, this result may simply derive from the greater power to detect recent sweeps that occurred after the divergence of the populations in question.

## Predominance of soft selective sweeps in recent human evolution

The central observation of Schrider and Kern was a dramatic excess of soft selective sweeps, which comprised 92.2% of all sweep signatures. Though rare overall, hard sweeps were relatively more common in non-African than in African populations, consistent with greater *N*_*e*_ in Africa as a result of the population bottleneck during the out-of-Africa migration. The widespread impacts of soft sweeps provide a strong argument against a model of mutation limitation, instead suggesting that the raw material necessary for adaptive evolution often segregates as standing variation at the time of selection onset. While potentially unexpected, given the low effective population size and genetic diversity of humans, Schrider and Kern [1] argue that these factors could be reconciled if the mutational target size—the number of sites at a given locus that when mutated produce an adaptive change in phenotype—is larger than previously assumed. An alternative explanation, that is not mutually exclusive, is that values of *N*_*e*_ are often estimated on the basis of standing levels of variation, which are highly sensitive to population bottlenecks and reflect longer time scales than are typically relevant for adaptation. If short-term *N*_*e*_ exceeds long-term *N*_*e*_, as would be expected in a recurrent bottleneck scenario, long-term *N*_*e*_ may greatly underestimate the availability of new mutations [9].

The list of candidate loci generated by Schrider and Kern [1] also provided an opportunity to test the enrichment of various gene annotations. Reassuring, but nonetheless interesting, was the observation that genes that are involved in spermatogenesis showed strong overrepresentation among candidate selected loci. This finding is consistent with data from diverse taxa showing that sperm-related genes experience rapid evolution in response to sperm competition, sexual conflict, and/or sexual selection. There was also enrichment for genes involved in central nervous system development and immune response, genes encoding virus-interacting proteins, and genes encoding proteins that physically interact with other proteins. Together, these findings may help in the generation of hypotheses about the phenotypes under selection and about the features of genetic architecture that constrain adaptation to particular gene sets.

## Pervasive effects of linked selection

Using S/HIC, Schrider and Kern [1] determined that approximately half of the genome is influenced, via linkage disequilibrium, by a nearby selective sweep. This finding has potentially wide-ranging implications for the dynamics of neutral and slightly deleterious variation. Indeed, under such a paradigm, deleterious mutations are expected to attain higher frequencies than predicted under mutation-selection-drift equilibrium. This hypothesis was borne out by the data, as moderate-frequency mutations that have been predicted to be deleterious by other methods were enriched in sweep-linked regions. More generally, a widespread influence of selective sweeps challenges the long-standing neutral theory of molecular evolution [10], which states that most variation within and between species does not impact fitness and is largely governed by random genetic drift. The limitations of this theory have long been recognized, but it has nevertheless served as an important framework for conceptualizing the dynamics of genetic variation while providing a useful null model to test for selection. If a large proportion of genetic variation is in fact influenced by linked positive selection, null models may need to be updated to better reflect this complexity.

## Open questions and next steps

Schrider and Kern [1] make a strong case for the idea that machine learning methods could be useful for addressing diverse questions in molecular evolution. Especially relevant to this field, machine learning is valuable for optimizing models with many tunable parameters that cannot be feasibly explored using other approaches. Furthermore, rather than manually defining features for classification, machine learning provides a principled framework for automatically inferring predictive features from the data. While these advantages are clearly attractive, machine-learning approaches should not be viewed as a panacea, given that they still rely on a training set. For evolutionary analyses, in which no ‘truth’ set exists, training sets are generally produced by simulation, which in turn depends on input parameters (e.g., demography, mutation rates, recombination rates) that are inferred from the data itself. In the specific case of positive selection, even sophisticated methods may be limited in their capacity to detect polygenic adaptation affecting complex traits, which in contrast to completed sweeps involves subtle changes in allele frequencies at many loci. Nevertheless, Schrider and Kern [1] provide an excellent example of how cutting-edge methods from computer science and statistics can be successfully brought to bear on long-standing questions in evolutionary biology. Furthermore, the list of candidate loci produced by their study provides abundant opportunities for detailed follow-up experiments to dissect the molecular-mechanistic basis of recent human adaptation.
